# Endocrine tumors of the gastrointestinal tract and pancreas:grading, tumor size and proliferation index do not predict malignant behavior

**DOI:** 10.1186/1746-1596-2-28

**Published:** 2007-08-08

**Authors:** Borislav A Alexiev, Cinthia B Drachenberg, John C Papadimitriou

**Affiliations:** 1 Department of Pathology, Division of Anatomic Pathology, University of Maryland Medical Center, 22 S. Greene Street, Baltimore, MD 21201-1595, USA

## Abstract

**Context:**

Gastrointestinal and pancreatic (GIP) endocrine tumors (ETs) have been regarded as slow growing neoplasms with distinct morphologic characteristics that behave less aggressively than carcinomas. The malignant potential of these tumors is difficult to predict.

**Objective:**

To evaluate prognostic parameters, namely tumor size, tumor grade, and Ki-67 index in relationship to metastatic behavior of GIP ETs.

**Design:**

Biopsies and surgical specimens from 38 patients with GIP ETs were selected. The study group comprised 16 males and 22 females (mean age 62.6 years; range 24–91). Formalin-fixed, paraffin-embedded tissue sections were stained with H&E, synaptophysin, chromogranin A, and Ki-67. Ki-67 index was evaluated using ChromaVision Automated Assisted Image Analysis software. Proliferative index was compared to tumor grade, and the degree of associations between tumor size, tumor grade, Ki-67 index and metastatic behavior of GIP ETs were evaluated.

**Results:**

Fifteen of the twenty-two (68.18%) surgically staged neoplasms presented with peritoneal dissemination, lymphogeneous, and/or hematogeneous metastases. Nine of the metastatic tumors were G1 (9/13, or 69.23%), 5 were G2 (5/7, or 71.42%), and 1 – G3 (1/2, or 50%). Overall, 10/15 (66.66%) metastatic tumors showed < 2% Ki-67 immunoreactivity. Four ileal ETs had a synchronous malignancy. No significant correlation was found to exist between tumor grade and Ki-67 index as well as between tumor size, tumor grade, Ki-67 index and metastatic behavior.

**Conclusion:**

The findings suggest that tumor size, tumor grade and Ki-67 index do not accurately predict malignant behavior of GIP ETs.

## Background

For many years, tumors of the disseminated endocrine system have been referred as "carcinoids" [1-3]. Oberndorfer coined this term in 1907 for these epithelial tumors in the gut that in general have a relatively monotonous structure and are less aggressive in their behavior than carcinomas [1-6]. Factors that determine the biologic behavior of endocrine tumors are complex and multifaceted. In the WHO classification of 2000, a distinction was made between well-differentiated endocrine tumors (which show benign behavior or uncertain malignant potential), well-differentiated endocrine carcinomas (which are characterized by less aggressive malignant behavior), and poorly differentiated endocrine carcinomas of high-grade malignancy [5-10]. However, the reproducibility of this grading system and its prognostic importance has sometimes been called into question [8]. In endocrine tumors, a number of clinicopathologic criteria proved to be useful predictors of malignant behavior; these include: site of origin; tumor type; tumor size; invasion of nearby tissue or deep wall invasion; angioinvasion and invasion of perineural spaces; presence of spotty necrosis; overt cellular atypia; more than two mitoses in 10 HPFs; Ki-67 index of more than 100/10 HPFs, or more than 2%; loss of chromogranin A immunoreactivity; and nuclear p53 accumulation [4-23]. However, the predictive value of such variables remains to be proven for tumors other than those of pancreas and stomach [6].

The objective of our study is to investigate the potential utility of cell proliferation (Ki-67 index) and histopathologic grading in augmenting the histological classification and assessing biologic aggressiveness in biopsies and surgical specimens with gastrointestinal and pancreatic (GIP) endocrine tumors (ETs).

## Methods

### Specimens

Thirty eight patients (22 women and 16 men, age range, 24–91 years, mean 62.4) were diagnosed as having a GIP ET (Table 1) at our institution between 2003 and 2005. Material was obtained from formalin-fixed, biopsies (n = 16) or resection specimens (n = 22). Hematoxylin-eosin-stained sections were available for review in all cases. The use of paraffin blocks for this study meets Institutional Review Board and Health Insurance Portability and Accountability Act requirements, and has been approved by the Institutional Review Board at the University of Maryland.

**Table 1:** Distribution of GIP ETs*

Location	Number of cases	Number of cases with metastatic disease
Stomach	3	0
Duodenum/upper jejunum	10	3/4**
Distal jejunum/ileum	10	7/9**
Appendix	2	0/2**
Colon	4	2/3**
Rectum	5	0
Pancreas	4	3/4**

* Gatrointestinal and pancreatic (GIP) endocrine tumors (ETs)

** Surgically staged tumors

### Classification and histopathologic grading

The tumors were classified according to the World Health Organization guidelines [5]. Histopathologic grading was performed according to the previously published criteria [5,7,8]. Briefly, grade 1 (G1) ETs were characterized by a variable structure, either with insular, trabecular, acinar, diffuse or mixed patterns, and by a monomorphic cytology with low atypia and rare if any mitosis (< 2/10 HPFs). Grade 2 (G2) ETs showed focal moderate cytologic atypia with few scattered mitotic figures (2–10/10 HPFs) and spotty necrosis. Grade 3 (G3) ETs demonstrated a solid growth pattern; the tumor cells were small, round, or oat-cell-like with marked nuclear pleomorphism, brisk mitotic activity (> 10/10 HPFs) and sizable areas of tumor necrosis.

### Immunohistochemical analysis

Immunostaining was performed according to the manufacturer's specifications. Briefly, four micron-thin sections were placed on the VentanaNexES autostainer (Ventana Medical Systems Inc, Tucson, Arizona) where they were treated with protease for 4 minutes and then incubated with prediluted anti-Ki-67 (Ventana, MM1, mouse monoclonal), anti-synaptophysin (CellMarque, rabbit polyclonal), and anti-chromogranin A (Ventana, LKZH10, mouse monoclonal) for 32 minutes. Recommended positive and negative controls were used. Visualization was performed using Ventana enhanced DAB detection kit. Ki-67 (MM1) stained slides were evaluated using ChromaVision Automated Quantitative Image Analysis software (objective, × 40). All tumor cell areas on the slide that stained positively were included as part of the evaluations regardless of the degree of staining. Cases with Ki-67 immunoreactivity of less than 1% were scored "0".

### Statistical analysis

The chi-square test was used to test the association between tumor grade and Ki-67 index as well as between tumor grade, tumor size, Ki-67 index and metastatic behavior of GIP ETs.

## Results

Of the 38 endocrine tumors, 29 were G1, 7 were G2, and 2 – G3. Fifteen of the twenty-two (68.18%) surgically staged neoplasms presented with peritoneal dissemination, lymphogeneous, and/or hematogeneous metastases (Table 2).

**Table 2:** Clinicopathologic data in GIP ETs*

Case No.	Location	Size	Grade	Ki-67	Metastases
1	Ileum	2.1 cm	G1	8%	Yes
2	Colon	11 cm	G3	55%	No
3	Colon	2.9 cm	G2	3%	Yes
4	Appendix	0.3 cm	G1	1%	No
5	Duodenum	N/A	G1	33%	N/A
6	Duodenum	N/A	G1	4%	N/A
7	Duodenum	1.3 cm	G2	4%	Yes
8	Pancreas	1.9 cm	G2	2%	Yes
9	Stomach	N/A	G1	6%	N/A
10	Duodenum	N/A	G1	1%	N/A
11	Colon	3.5 cm	G2	0%	Yes
12	Rectum	N/A	G1	0%	N/A
13	Duodenum	2.5 cm	G1	0%	Yes
14	Duodenum	N/A	G1	0%	N/A
15	Duodenum	N/A	G1	3%	N/A
16	Rectum	N/A	G1	0%	N/A
17	Rectum	N/A	G1	0%	N/A
18	Colon	N/A	G1	0%	N/A
19	Stomach	N/A	G1	3%	N/A
20	Duodenum	N/A	G1	1%	N/A
21	Ileum	1.3 cm	G1	3%	No
22	Ileum	N/A	G1	1%	N/A
23	Ileum	0.9 cm	G1	0%	Yes
24	Stomach	N/A	G1	2%	N/A
25	Ileum	3.0 cm	G1	0%	Yes
26	Rectum	N/A	G1	1%	N/A
27	Ileum	0.7 cm	G1	1%	Yes
28	Duodenum	N/A	G1	3%	N/A
9	Pancreas	16 cm	G3	32%	Yes
30	Pancreas	4.1 cm	G2	2%	No
31	Ileum	1.4 cm	G1	1%	No
32	Ileum	2.4 cm	G1	0%	Yes
33	Duodenum	1.5 cm	G2	4%	No
34	Pancreas	2.8 cm	G1	3%	Yes
35	Duodenum	4 cm	G2	1%	Yes
36	Ileum	1.2 cm	G1	2%	Yes
37	Ileum	2.5 cm	G1	0%	Yes
38	Appendix	1 cm	G1	6%	No

* Gastrointestinal and pancreatic (GIP) endocrine tumors (ETs)

**Figure 1:**
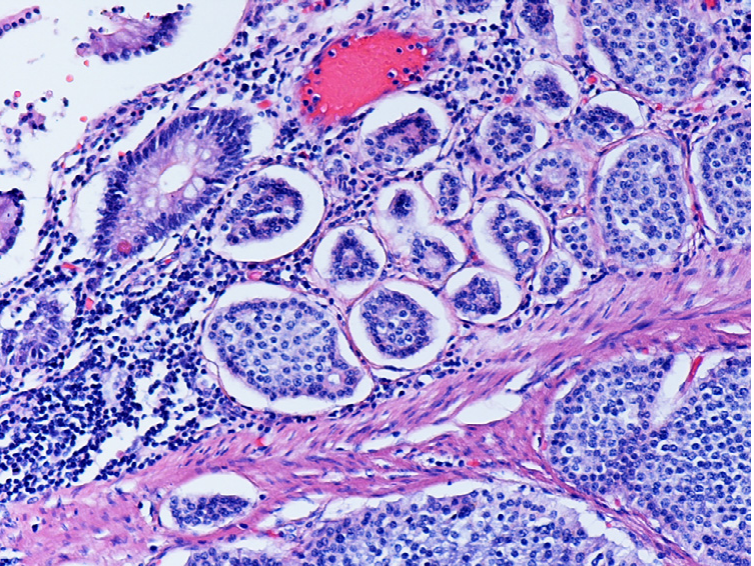
Metastatic, G1 endocrine tumor involving ileal mucosa and submucosa. Note insular growth pattern. Hematoxylin and eosin. × 200.

**Figure 2:**
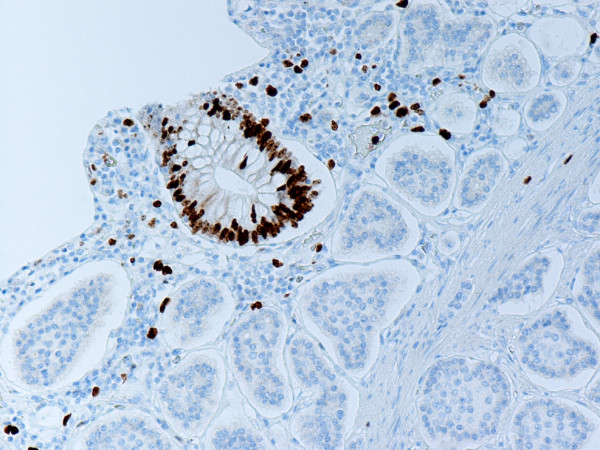
Same tumor as shown in Fig. 1. Ki-67 monoclonal antibody stains nuclei of crypt epithelial and stromal cells. The tumor cells are negative. Anti-Ki-67. × 200.

**Figure 3:**
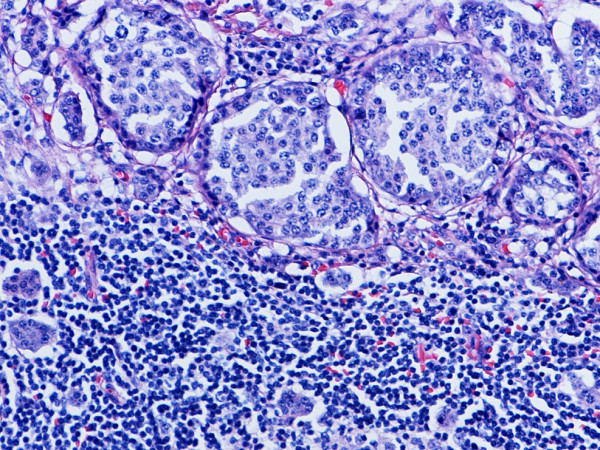
Lymph node metastasis from G1 endocrine tumor as shown in Fig. 1. Hematoxylin and eosin. × 200.

**Figure 4:**
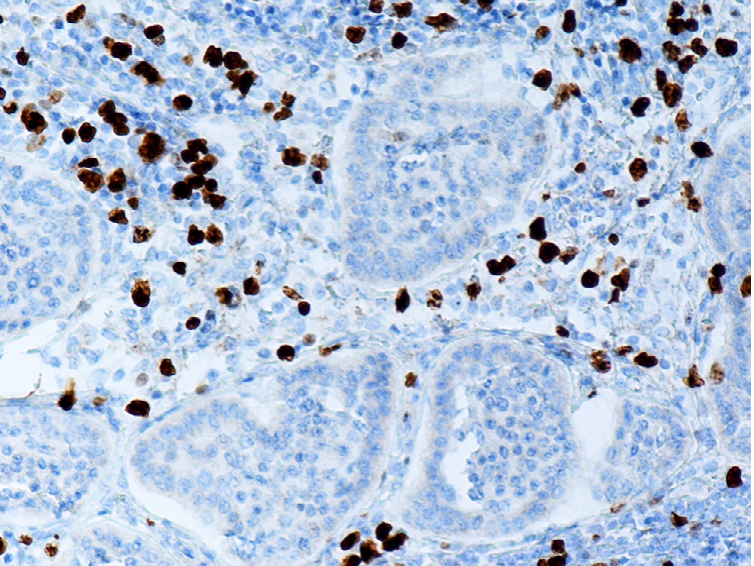
Lymph node metastasis from G1 endocrine tumor as shown in Fig. 1. Ki-67 monoclonal antibody stains nuclei of lymphocytes. The tumor cells are negative. Anti-Ki-67. × 200.

**Figure 5:**
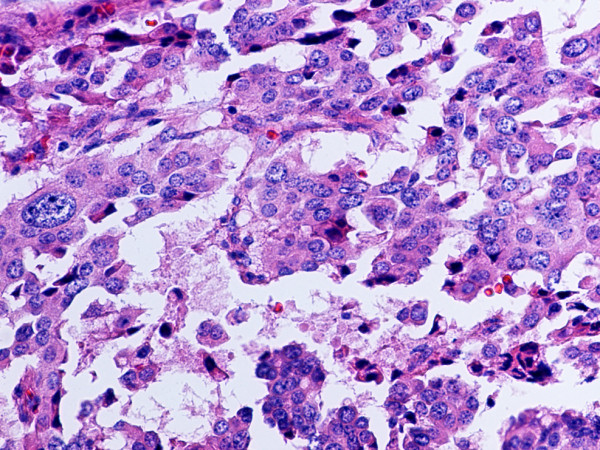
Metastatic, G2 endocrine tumor. Note anisonucleosis and focal tumor necrosis. Hematoxylin and eosin. × 400.

**Figure 6:**
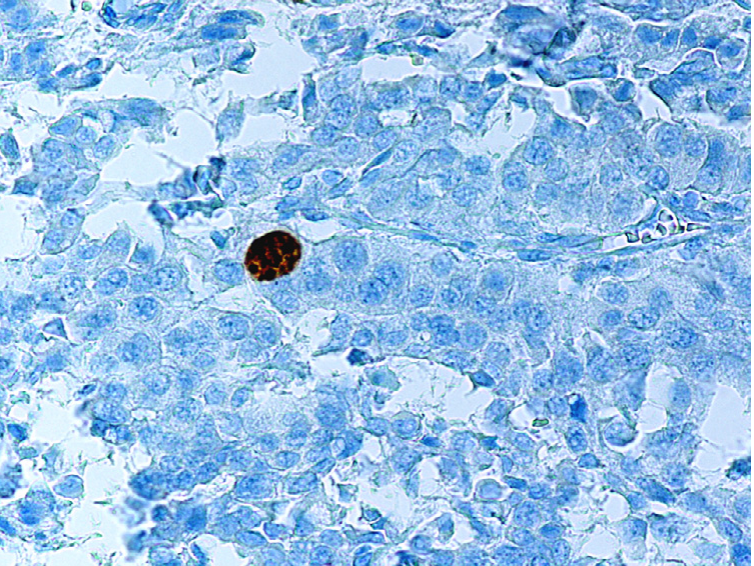
Same tumor as shown in Fig. 5. Only one tumor cell nucleus stains positive with Ki-67 monoclonal antibody. Anti-Ki 67. × 400.

**Figure 7:**
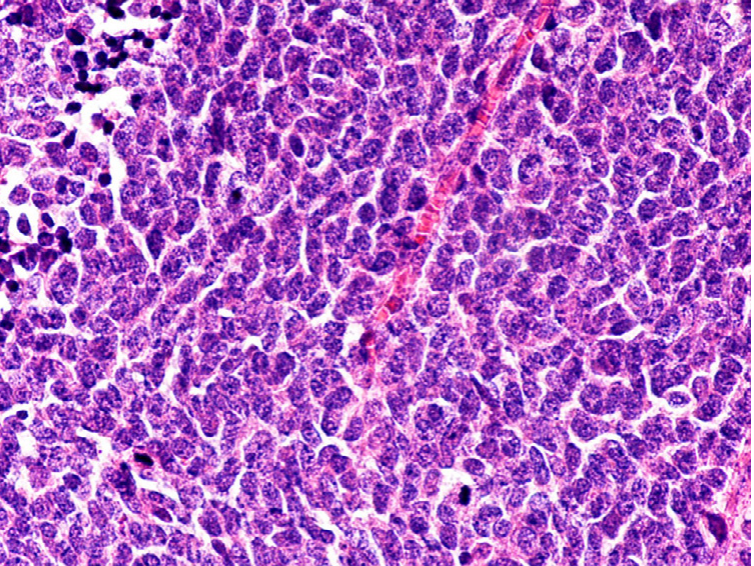
Non-metastatic, G3 endocrine tumor. Hematoxylin and eosin. × 400.

**Figure 8:**
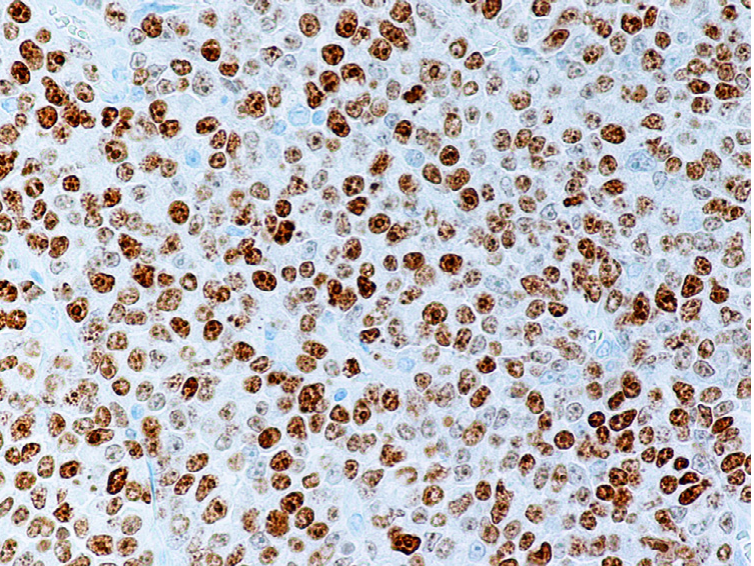
Same tumor as shown in Fig. 7. Numerous tumor cell nuclei stain positive with Ki-67 monoclonal antibody. Anti-Ki-67. × 400.

Nine of the metastatic tumors were G1 (9/13, or 69.23%), 5 were G2 (5/7, or 71.42%), and 1 – G3 (1/2, or 50%) (Table 3). Unexpectedly high association with metastasis was found in small (< 2 cm), G1, endocrine tumors (Table 2). The results of the automated quantitative Ki-67 immunoreactivity analysis in comparison to tumor grade and metastatic disease are shown in Tables 4 and 5. Overall, 10/15 (66.66%) metastatic tumors showed < 2% Ki-67 immunoreactivity (Figs. 1, 2, 3, 4, 5, 6). On the other hand, non-metastatic G1-3 ETs demonstrated a high Ki-67 index (Figs. 7 and 8) (Table 2). No statistically significant correlation was found to exist between tumor grade, tumor size, Ki-67 index and metastatic behavior (Tables 3, 5 and 6).

**Table 3:** Correlation between tumor grade and metastatic disease

Grade	Metastases (+)	Metastases (-)	Total
G1	9	4	13
G2	5	2	7
G3	1	1	2

Total	15	7	22

Degrees of freedom: 2

Chi-square = 0.345368916797488

For significance at the .05 level, chi-square should be greater than or equal to 5.99.

The distribution is not significant.

***p ***is less than or equal to 1.

**Table 4:** Correlation between tumor grade and Ki-67 index

Grade	Ki-67 Index > 2%	Ki-67 Index < 2%	Total
G1	10	19	29
G2	3	4	7
G3	2	0	2

Total	15	23	38

* Cases with Ki-67 index of 2% were included in the < 2% category.

Degrees of freedom: 2

Chi-square = 3.40255586492468

For significance at the .05 level, chi-square should be greater than or equal to 5.99.

The distribution is not significant.

***p ***is less than or equal to 0.20.

**Table 5:** Correlation between Ki-67% index and metastatic disease

Ki-67 Index	Metastases (+)	Metastases (-)	Total
> 2%	5	4	9
< 2%*	10	3	13

Total	15	7	22

* Cases with Ki-67 index of 2% were included in the < 2% category.

Degrees of freedom: 1

Chi-square = 1.11925111925112

For significance at the .05 level, chi-square should be greater than or equal to 3.84.

The distribution is not significant.

***p ***is less than or equal to 1.

**Table 6:** Correlation between tumor size and metastatic disease

Size	Metastases (+)	Metastases (-)	Total
< 2 cm*	5	5	10
> 2 cm	10	2	12

Total	15	7	22

* Tumors measuring 2 cm were includes in < 2 cm category.

Degrees of freedom: 1

Chi-square = 2.79365079365079

For significance at the .05 level, chi-square should be greater than or equal to 3.84.

The distribution is not significant.

***p ***is less than or equal to 0.10.

Four of the 22 surgically staged GIP ETs had a synchronous malignancy: one non-metastatic ileal ET with synchronous non-metastatic colon adenocarcinoma, and three metastatic ileal ETs with synchronous metastatic colon and common bile duct adenocarcinomas, and non-metastatic renal cell papillary carcinoma. Both ETs, although smaller in size, showed a higher metastasis rate than the synchronous metastatic adenocarcinomas.

## Discussion

For many years, GIP ETs have been regarded as slow growing neoplasms with distinct morphologic characteristics that behave less aggressively than conventional adenocarcinomas [1]. The malignant potential of endocrine tumors is difficult to predict. In this context, the latest WHO classification provides a useful framework for the evaluation of clinicopathological and functional properties of these neoplasms [6]. However, a disadvantage of the current WHO classification system is that it is not possible to evaluate some of the well known criteria for malignancy, namely the presence of metastases, and deep wall invasion or invasion of nearby tissue in biopsy specimens. Recently, attempts have been made to define histological and immunohistochemical prognostic factors that may aid in predicting the biologic behavior of GIP ETs in the context in which they commonly present to the surgical pathologist. In this context, the study by Hochwald et al [24] affirmed the clinical usefulness of a two-tiered classification of differentiated pancreatic endocrine neoplasms into low- and intermediate-grade groups on the basis of tumor necrosis (absent or present) and mitotic rate (< 2 mitoses/50 HPFs, or > 2 mitoses/50 HPFs) [24]. Moreover, high-grade ETs were defined as neoplasms characterized by a solid growth pattern, cytologic atypia, > 10 mitoses/10 HPFs (Ki-67 index > 10%), and widespread necrosis [6,7,24].

This study investigated the histological grading, Ki-67 index, tumor size, and metastatic behavior in a group of patients with GIP ETs. The goal was to evaluate Ki-67 index using ChromaVision Automated Image Analysis software, and to determine whether histological grade, tumor size, and Ki-67 index had any bearing on metastatic behavior. We observed unexpectedly high aggressiveness (multiple lymphogenous and hematogenous metastases, and peritoneal implants) in small (< 2 cm), low-grade ETs, with low Ki-67 index. These observations are consistent with other reports [16,18,25]. In his excellent study, based on analysis of 1914 reported cases with gastrointestinal endocrine tumors, Soga [25] found a high aggressiveness in metastasis rates in both rectal and gastric small carcinoids exhibiting values significantly higher than those of small carcinomas. We did not find statistically significant correlation between tumor grade and Ki-67 index, as well as between tumor grade, tumor size, Ki-67 index and metastatic behavior of GIP ETs. These observations are in disagreement with earlier positive findings [8,11,12,15,19]. This disagreement might be explained by methodological differences, or the different antibodies employed. In this context, ChromaVision Automated Ki-67 index analysis provides superior accuracy in comparison to semi quantitative evaluation of Ki-67 positivity. Most importantly, this study shows the limitations of the current WHO classification in assessment of the metastatic behavior of GIP ETs. Thus, we were able to show that small, low-grade ETs, with low proliferative index, which met the criteria of the WHO classification criteria for benignity, behaved in a highly aggressive fashion. On the other hand large, intermediate- and high-grade, with high proliferative index ETs, which met the WHO classification criteria for malignancy, behaved in a benign fashion, i.e. without metastatic disease. Currently, we cannot explain the highly aggressive behavior of small, low-grade, with low proliferative index ETs. Previous studies suggest that tumors with a short cell cycle may grow rapidly but without necessarily manifesting numerous mitotic figures at any moment [8]. In addition, recent reports indicate that nuclear survivin and valosin-containing protein (p97) are useful prognostic factors in ETs [26,27].

The observed increased risk of synchronous malignancies in GIP ETs is consistent with previous reports [28-30]. The results illustrate the need for a thorough search for additional neoplasms in patients with ileal ETs.

## Conclusion

In conclusion, the results suggest that tumor grade does not significantly correlate with Ki-67 index. Further, tumor grade, tumor size, and Ki-67 index do not accurately predict malignant behavior of GIP ETs.

## Competing interests

The author(s) declare that they have no competing interests.

## Authors' contributions

BAA evaluated the H&E and immunohistochemical stains, confirmed the diagnosis, designed the report and drafted the manuscript. CID and JCP provided consultation.

All authors read and approved the final manuscript.
